# Role of living donor liver transplantation in the treatment of hepatitis C virus infection

**Published:** 2011-06-01

**Authors:** Georgios Tsoulfas, Polyxeni Agorastou

**Affiliations:** 1 Department of Surgery, Aristoteleion University of Thessaloniki, Thessaloniki, Greece; 2 Department of Gastroenterology, Aristoteleion University of Thessaloniki, Thessaloniki, Greece

**Keywords:** Hepatitis C virus, Living donor, Liver transplantation, Liver cirrhosis, Treatment

## Abstract

Hepatitis C virus (HCV) infection is one of the most common indications for liver transplantation worldwide. Because of the existing organ shortage, adult-to-adult living donor liver transplantation (LDLT) has become an important method of expanding the donor pool to meet the ever-increasing need. However, despite advantages such as the quality of the hepatic graft and the timing of the transplant, the exact role of LDLT in the treatment of HCV is still unclear. In this review, we aim to address some of these issues in an effort to highlight both the advantages and disadvantages, as well as to identify the main challenges, of using LDLT for treating patients with HCV infection.

## 1. Introduction

Cirrhosis due to chronic hepatitis C virus (HCV) infection is one of the leading indications for liver transplantation (LT) worldwide. Studies have shown that 75-85% of individuals infected with HCV develop chronic infection, which persists for at least 6 months after onset, with the rate of chronic infection varying by age, gender, race, and immune system status[1]. Long-term infection has been associated with serious clinical sequelae, including the development of hepatic fibrosis, cirrhosis of the liver, portal hypertension, and hepatocellular carcinoma (HCC)[2][3][4]. Although the natural history of HCV infection is believed to be variable, it is estimated that up to 20% of chronically infected individuals will develop liver cirrhosis over a 20- to 25-year period and that these individuals are at increased risk of developing HCC [2][4]. The magnitude of the impact of HCV becomes more evident if we consider that in the USA, there are nearly 10,000 deaths annually due to HCV-related diseases, that HCV is responsible for nearly half of all HCC cases, and that the risk of developing HCC after the onset of cirrhosis is 3-4% per year [5]. As a result, HCV-cirrhosis, which accounts for 35-40% of all cases of cirrhosis, has become the most common indication for LT in the USA [6].

Unfortunately, the increased need for livers is exacerbated by an organ shortage. In an effort to expand the limited donor pool, there has been increased focus on living donor liver transplantation (LDLT). In general, individuals with decompensated cirrhosis who meet the standard indications for LT, do not have any contraindications, and have a Model for End-stage Liver Disease (MELD) score of 15 or higher are appropriate candidates for LDLT. Patients with a lower MELD score would not benefit from any form of LT, whereas those with a significantly higher MELD score are potentially too sick to justify the use of a living donor liver graft [7]. The problem, however, remains that the number of LDLT surgeries being conducted is low. One explanation for this is that the strenuous process that all potential living donors have to go through leads to a high rate of attrition among donors. In a report from 1 center, there was a 50% rate of attrition, mainly because of medical co-morbidities, psychosocial factors, financial issues, and the availability of an organ from a deceased donor during the evaluation process [8]. Given the fact that in most centers only about one-third of the patients on the list may have an available living donor and of these, no more than half may undergo the evaluation successfully, only about 15% of patients on the list have the option of a LDLT [9]. A second explanation is the severity of the donor surgery. Although living donors have an overall perception that donation is a positive event they do not regret participating in, with very few durable side effects, it is still a surgery that may lead to complications in 20-40% of donors and carries a mortality risk of 0.3-0.5% for the donor [10][11]. Two highly publicized donor deaths led to a significant drop in the number of centers in the USA performing the procedure and the number of LDLT cases between 2001 and 2003 [12][13][14].

Overall, it is clear that the relationship between end-stage liver disease secondary to HCV and LDLT is one of necessity. LDLT is a tremendous tour de force, both technically and ethically, presenting several challenges to the medical community because it is a unique procedure in which healthy people undergo a high-risk operation that has no benefit to their health. HCV infection is a leading indication for LT, and in the face of organ shortage, every effort should be made to expand the donor pool to meet the needs of the patients with HCV infection. In addition, patients with HCC usually have a low MELD score, as their main problem and more imminent threat is not one of hepatic insufficiency, but rather one of advancing cancer. For these patients, the option of LDLT presents an opportunity for a timely cure. As a result, the need for living donation is unlikely to decrease any time soon, and thus, it is important to determine the parameters for its proper role in the treatment of HCV infection.

## 2. LDLT for HCV: Issues and challenges

2.1. Graft quality

Compared to a full-sized deceased donor organ, a living donor allograft has significantly less hepatic mass; this finding has led to the suggestion that the living donor allograft should be treated as an "extended donor criteria" organ [15]. A reason for concern is the small-for-size syndrome, characterized by synthetic dysfunction, elevated aminotransferases, and prolonged cholestasis[16]. Small-for-size syndrome may resolve with supportive care and time, with transaminases returning to normal within days, but cholestasis can take weeks to resolve. The problem is that an allograft with transaminitis and cholestasis is particularly vulnerable to the acidosis, hypoglycemia, renal insufficiency or failure, and infections that may occur in the immediate postoperative period, leading to potentially irreversible damage that may prove deadly without retransplantation. It should also be stressed that small-for-size syndrome is not solely the result of transplanting a smaller volume graft, but is also the direct result of graft hemodynamics, as excessive portal inflow, combined with compromised venous drainage of the partial graft, can lead to overperfusion and decreased function of the allograft [17][18][19].

These valid concerns are outweighed by the significantly lower cold ischemia time of the living donor allograft than the deceased donor organ, as well as the fact that the donor is a healthy, extensively screened individual [20]. Assessment of potential donors includes both medical and psychosocial evaluations, performed by separate medical teams, to ensure that the donor is fully informed of the potential risks to themselves and the alternatives that the recipient may have in the event that the living donation does not proceed. The option to stop the process at any time is given in a way that would not affect the relationship of the donor with the recipient. The third portion of the evaluation involves the anatomical assessment in which the quality, quantity, and anatomy of the donor's liver is considered. This thorough procedure leads to results at least as good as those achieved with organs from deceased donors, as we will see later in the paper.

2.2. Timing of transplantation

Determining the appropriate timing for an LT, particularly for patients with HCV infection, requires a balancing act. In particular, the recipient has to be healthy enough to undergo the LT safely, but on the other hand sick enough so that the morbidity and mortality associated with the procedure do not outweigh the benefits [21]. This is even more critical for patients with HCV infection, in whom being able to avoid premature transplant allows a delay in the recurrence of HCV in the new graft, which may prolong the recipient's life, as well as allow time for the development of improved antiviral therapy. The principal advantage of LDLT is that it allows the transplant team to choose the proper timing, thereby decreasing the risk of decompensation or death of a patient while on the waiting list, as well as providing flexibility, which can allow an attempt at pretransplant viral eradication [22][23]. If it is possible to proceed to the transplant with a recipient negative for serum HCV RNA on therapy, then the percentage of posttransplant HCV recurrence after LDLT is very low (10%) and could essentially constitute a cure for HCV infection through transplantation[24]. Furthermore, it is easier for a patient with a lower MELD score to tolerate a full-dose regimen for HCV eradication, and once viral eradication is achieved, one can proceed to the LDLT and achieve the optimal result. This approach could potentially cure about 40% of the individuals with HCV who undergo LDLT [25].

2.3. Results of LDLT for HCV

Comparable data between LDLT and deceased donor liver transplantation (DDLT) for HCV have been reported using the UNOS database [26]. In this large study comparing transplant recipients with chronic HCV who received an LDLT (No. = 279) to those who received a DDLT (No. = 3955), the one-year survival rate was 87% in both groups and 2-year survival rate was 83% and 81% in the LDLT and DDLT groups, respectively (p = 0.68). Several other studies have shown similar results, either using UNOS data or single, large center experience, and demonstrated no negative impact of LDLT on the results of liver transplantation for HCV infection [27][28][29]. The Adult-to-Adult Living Donor Liver Transplantation Cohort Study (A2ALL), a multicenter study of 275 liver transplants (181 LDLT and 94 DDLT) is one of the largest studies from which conclusions can be drawn [30]. This study showed an overall statistically significant survival advantage for DDLT than for LDLT (82% vs. 74% at 3 years). However, a previous study showed poorer results when patients were separated into 3 groups: the cases of DDLT, the first 20 cases of LDLT performed at the center, and the remaining LDLT cases[31]. In this study, although DDLT was more advantageous than LDLT (in the group of the first 20 LDLT cases), there was no difference in survival or rate of progression to fibrosis between the DDLT and the later LDLT cases. This raised the issue of center experience and the effect of the learning curve, as LDLT poses certain unique technical challenges such as vascular problems, biliary complications, and small-for-size syndrome [32][33]. Allowing for the importance of center experience may mean that LDLT for HCV infection may be at least as safe as DDLT. This still leaves the question of increased and more aggressive recurrence of HCV infection after LDLT than after DDLT.

2.4. HCV recurrence after LDLT

LDLT grafts have tremendous growth potential, as the graft regenerates 150,000 hepatocytes every second in the first week after transplantation and doubles in size within 4 weeks [34][35]. Although this is beneficial in restoring the necessary hepatocyte mass for the patient, it raises concerns regarding the effect that it may have on viral replication and the development of cholestatic hepatitis, a rapidly progressive and virulent form of HCV infection. Factors believed to work in favor of decreased HCV recurrence in LDLT than in DDLT include less acute cellular rejection with less immunosuppression; younger, healthier recipients; fewer African-American LDLT recipients; lower HCV posttransplantation viral load; and the opportunity for pretransplantation treatment to eradicate HCV or reduce the viral load [36][37][38]. The factors that may be responsible for the more aggressive recurrence pattern sometimes seen with LDLT include increased HLA matching of the recipient with the donor, especially since a significant number are relatives, and the very active hepatocyte regeneration, leading to increased intrahepatocyte HCV proliferation [39][40]. Moreover, experimental data suggest that liver regeneration induces low-density lipoprotein receptor expression, which might facilitate HCV entrance into the hepatocytes [41][42].

While 2 large studies have shown a similar incidence and severity of HCV recurrence between LDLT and DDLT recipients, a third study found that the incidence of cholestatic hepatitis is significantly greater in LDLT recipients[26][43][44][45]. In an effort to resolve this discrepancy, a careful comparison of protocol liver biopsies from 23 LDLT and 53 DDLT recipients did not reveal significant differences in the degree of hepatic inflammation between the 2 groups over 3 years, and similar amounts of fibrosis in the LDLT group[43].

2.5. Treatment for HCV recurrence after LDLT

The fact that HCV may recur earlier and, potentially, in a more aggressive form after LDLT means that strategies for HCV recurrence treatment are crucial. Treating DDLT-candidate patients with HCV infection is not usually feasible before the transplant to achieve viral resolution because the patients cannot tolerate full-dose treatment with ribavirin and interferon, given their state of hepatic dysfunction. The alternative, aggressive, preemptive treatment after LT has not shown great success [46][47]. LDLT has a distinct advantage over DDLT in the treatment of HCV recurrence because it is possible to treat recipients for HCV infection aggressively prior to the transplant, as they are more stable and can tolerate treatment. In addition, it is possible to arrange the timing of the transplant, so that it proceeds as soon as viral clearance is achieved. This has been shown to lead to a sustained virological response, and is thus the most definitive way to address the issue of recurrence[48].

2.6. Immunosuppression in LDLT for HCV infection

Another key issue in dealing with the conundrum of HCV recurrence after LT is the choice of immunosuppression. Standard posttransplant immunosuppression consists of a calcineurin inhibitor (cyclosporine or tacrolimus), a tapering dose of corticosteroids, and in the majority of cases, an antiproliferating agent for lymphocytes (mycophenolate mofetil or azathioprine). Antibodies to T cells (antithymocyte globulin) or to the interleukin-2 receptor (basiliximab) are less often used as induction agents, to either delay the initiation of the calcineurin inhibitor to protect renal function, or to proceed with a very rapid steroid taper. Although clear data in favor of a single baseline immunosuppression regimen are limited, there is an agreement that more intense immunosuppressive regimens can lead to worse outcomes. That is, patients receiving high bolus steroids and induction therapies in the form of antibodies to lymphocytes or interleukin-2 receptor are more likely to encounter HCV-induced graft failure and undergo a rapid progression to cirrhosis secondary to cholestatic hepatitis[49][50]. The problem is that these agents are also used to treat rejection, which has been associated with decreased survival in patients with HCV infection [51]. The difficulties become even more apparent if we consider that differentiating between HCV recurrence and acute rejection on the basis of a biopsy examination may not be straightforward, as both have an element of portal inflammation and there is significant overlap.

The above-mentioned issues in finding the optimal immunosuppression regimen for patients with HCV infection after LT are even more evident in the case of LDLT in which rejection is more prevalent. This leads to a balancing act between finding the appropriate amount of immunosuppression to avoid rejection on one hand, and avoiding uncontrolled recurrence of HCV on the other hand[52]. The main destabilizing factors are rapid changes in the level of immunosuppression, which leads to intense viral replication. Several strategies, such as rapidly tapering steroids or steroid-free immunosuppression with or without induction antibodies, have been employed to achieve this balance. However, stemming from experience, the most sound practice appears to be the attainment of adequate immunosuppression to avoid the incidence of rejection, and treating any episodes that may occur with gradual increases in the existing regimen, rather than using bolus steroids or antibodies [53].

2.7. Retransplantation and LDLT for HCV infection

The accelerated recurrence of HCV infection after LT raises the issue of whether to retransplant patients with graft failure. Results for retransplantation for HCV recurrence have been discouraging overall [54][55][56]. Although many studies have demonstrated that HCV-positive retransplant recipients have worse survival rates than HCV-negative recipients, there is conflicting data regarding whether or not the cause of allograft failure in HCV-positive patients influences survival. Most studies have actually demonstrated that recurrent HCV as a cause of allograft failure is less common and that allograft loss in HCV-positive recipients is much more likely to be caused by a nonfunctioning allograft, hepatic artery thrombosis, and chronic rejection [57][58][59]. Most data suggest that survival after retransplantation is poor in patients with HCV infection, even in those retransplanted for non-HCV-related indications [57][60][61].

To deal with this problem, arguments ranging from performing retransplantation for HCV-induced allograft failure early in its disease course to refusing retransplantation to patients infected with HCV because it is unnecessary and futile have been forwarded [56][60][62]. This is exactly where the use of LDLT raises important ethical and practical issues and offers certain possibilities with regard to the treatment of HCV-positive recipients. In particular, LDLT would not deplete the donor organ pool and would lead to the use of scarce deceased donor organs by patients who are awaiting primary liver transplantation. Despite inferior outcomes, a better tactic may be to consider retransplantation for recurrent HCV in those patients whose primary transplant was an LDLT, as the initial allograft did not deplete the donor pool.

2.8. HCC and LDLT

Patients with HCV infection have a higher rate of HCC, and since a pretransplant diagnosis of HCC has been shown to be an independent predictor of reduced overall patient survival beyond 90 days, timely LT is of the outmost importance [30]. The most effective approach to reduce the dropout rate on the LT waiting list is to expand the number of available livers. A primary strategy towards this goal is the use of LDLT. Decision analyses, taking into account the risk of dropout while on the waiting list (4% per month), the expected survival of the recipient using the Milan criteria (70% at 5 years), and the risk for the donor (0.3-0.5% mortality), suggest that this is a cost-effective approach if the wait time exceeds 7 months [63]. Recently, MELD exception points for HCC were modified, as new data showed that former prioritization points for HCC were unfairly favoring access to DDLT for these patients. Compared to DDLT, LDLT offers the advantage of timely access to LT, while at the same time preserving the equity principle by not depleting the donor pool. Additionally, the development of live donation has stimulated discussion about the expansion of the tumor burden limits for HCC patients. Since transplantation can be performed in a timely manner and with recent staging, there have been proposals that LDLT may be an option for patients whose tumor stage does not allow listing for DDLT. Deceased donor livers would then be allocated to patients with the best potential outcome (within Milan criteria and survival of 70% at 5 years), and living donation livers would benefit patients with a lower survival. Although this has a sound theoretical basis, data are not yet available to support utilizing such expanded criteria[64]. Furthermore, this policy of using LDLT for HCC patients outside the currently accepted criteria raises the question of what would happen if these patients require retransplantation. Overall, LDLT remains an important alternative for patients with HCV infection and HCC of small size, as it provides access to LT in a timely manner.

2.9. Economic aspects of LDLT for HCV infection

In current times of fiscal constraint in most countries worldwide, any evaluation of a treatment ultimately requires that society consider the financial burden associated with that treatment. Several studies have attempted to evaluate the extensive resources required for LDLT. A single center in New York found no increase in resources by examining the billing data[65]. As financial cost has many different faces, a study using the A2ALL outcome data showed that although the cost increased with LDLT, the survival of patients awaiting transplantation also increased[66]. An increase of 0.5 quality-adjusted life years resulted from being on a waiting list, with the possibility of receiving both deceased and live donation, than from being on a waiting list with only the possibility of deceased donation. The cost of a transplant from the latter list was on average $151,613, whereas that from a list with both options was $208,149. Although LDLT represents a higher immediate cost, we must consider that there are factors that will counterbalance this cost. These include the learning curve that leads to the performance of LDLTs with fewer complications and quicker return to full activity for the donors, as well as the fact that we are able to transplant patients before they become too sick, thus affording a quicker recovery for the recipient. Additional comprehensive outcomes studies are needed to obtain a more detailed picture of the financial aspects of LDLT.

## 3. Conclusion

The outcomes and patient survival after LDLT for treatment of HCV infection appear to be comparable to those of patients undergoing DDLT. The main advantage for the recipient is a decrease in the waiting time, which can prove to be life saving. From a global perspective, the advantage becomes even more evident in countries with no history of DDLT and in which LDLT has proven to be an excellent way to increase the donor pool. However, concerns remain about the problem of aggressive HCV recurrence, as well as the safety of the donor. That these concerns have led a great number of centers to decide against retransplantation for patients with HCV recurrence, makes LDLT all the more important. This is because LDLT does not deplete the deceased donor pool, and hence, these patients should not be excluded if retransplantation is necessary. In addition, the continuous increase in the number of patients with HCV infection worldwide means that this group of patients that present a challenge to the health system cannot and must not be ignored, irrespective of the difficulties in the management of their condition. It is imperative that these issues are addressed in a multicenter effort, such as the A2ALL study, and with careful long-term follow-up.

## References

[1] Hoofnagle JH (2002). Course and outcome of hepatitis C. Hepatology.

[2] (2002). NIH Consensus Statement on Management of Hepatitis C: 2002. NIH Consens State Sci Statements.

[3] Hoofnagle JH (1997). Hepatitis C: the clinical spectrum of disease. Hepatology.

[4] Strader DB, Wright T, Thomas DL, Seeff LB (2004). Diagnosis, management, and treatment of hepatitis C. Hepatology.

[5] Davis GL, Albright JE, Cook SF, Rosenberg DM (2003). Projecting future complications of chronic hepatitis C in the United States. Liver Transpl.

[6] Szabo E, Lotz G, Paska C, Kiss A, Schaff Z (2003). Viral hepatitis: new data on hepatitis C infection. Pathol Oncol Res.

[7] Freeman RB (2003). The impact of the model for end-stage liver disease on recipient selection for adult living liver donation. Liver Transpl.

[8] Trotter JF, Wachs M, Trouillot T, Steinberg T, Bak T, Everson GT, Kam I (2000). Evaluation of 100 patients for living donor liver transplantation. Liver Transpl.

[9] Rudow DL, Russo MW, Hafliger S, Emond JC, Brown RS, Jr (2003). Clinical and ethnic differences in candidates listed for liver transplantation with and without potential living donors. Liver Transpl.

[10] Castedal M, Andersson M, Polanska-Tamborek D, Friman S, Olausson M, Fehrman-Ekholm I (2010). Long-term follow-up of living liver donors. Transplant Proc.

[11] Trotter JF, Wachs M, Everson GT, Kam I (2002). Adult-to-adult transplantation of the right hepatic lobe from a living donor. N Engl J Med.

[12] Brown RS,Jr, Russo MW, Lai M, Shiffman ML, Richardson MC, Everhart JE, Hoofnagle JH (2003). A survey of liver transplantation from living adult donors in the United States. N Engl J Med.

[13] Miller C, Florman S, Kim-Schluger L, Lento P, De La Garza J, Wu J, Xie B, Zhang W, Bottone E, Zhang D, Schwartz M (2004). Fulminant and fatal gas gangrene of the stomach in a healthy live liver donor. Liver Transpl.

[14] Russo MW, Brown RS,Jr (2004). Adult living donor liver transplantation. Am J Transplant.

[15] Renz JF, Kin C, Kinkhabwala M, Jan D, Varadarajan R, Goldstein M, Brown RJr, Emond JC (2005). Utilization of extended donor criteria liver allografts maximizes donor use and patient access to liver transplantation. Ann Surg.

[16] Tucker ON, Heaton N (2005). The 'small for size' liver syndrome. Curr Opin Crit Care.

[17] Gonzalez HD, Liu ZW, Cashman S, Fusai GK (2010). Small for size syndrome following living donor and split liver transplantation. World J Gastrointest Surg.

[18] Kiuchi T, Kasahara M, Uryuhara K, Inomata Y, Uemoto S, Asonuma K, Egawa H, Fujita S, Hayashi M, Tanaka K (1999). Impact of graft size mismatching on graft prognosis in liver transplantation from living donors. Transplantation.

[19] Shimada M, Ijichi H, Yonemura Y, Harada N, Shiotani S, Ninomiya M, Terashi T, Yoshizumi T, Soejima Y, Suehiro T, Maehara Y (2004). The impact of splenectomy or splenic artery ligation on the outcome of a living donor adult liver transplantation using a left lobe graft. Hepatogastroenterology.

[20] Gali B, Rosen CB, Plevak DJ (2011). Living Donor Liver Transplantation: Selection, Perioperative Care, and Outcome. J Intensive Care Med.

[21] Merion RM, Schaubel DE, Dykstra DM, Freeman RB, Port FK, Wolfe RA (2005). The survival benefit of liver transplantation. Am J Transplant.

[22] Liu CL, Lam B, Lo CM, Fan ST (2003). Impact of right-lobe live donor liver transplantation on patients waiting for liver transplantation. Liver Transpl.

[23] Russo MW, LaPointe-Rudow D, Kinkhabwala M, Emond J, Brown RS,Jr (2004). Impact of adult living donor liver transplantation on waiting time survival in candidates listed for liver transplantation. Am J Transplant.

[24] Everson GT (2003). Treatment of patients with hepatitis C virus on the waiting list. Liver Transpl.

[25] Everson GT (2005). Should we treat patients with chronic hepatitis C on the waiting list?. J Hepatol.

[26] Bozorgzadeh A, Jain A, Ryan C, Ornt D, Zand M, Mantry P, Lansing K, Orloff M (2004). Impact of hepatitis C viral infection in primary cadaveric liver allograft versus primary living-donor allograft in 100 consecutive liver transplant recipients receiving tacrolimus. Transplantation.

[27] Rodriguez-Luna H, Vargas HE, Sharma P, Ortiz J, De Petris G, Balan V, Byrne T, Moss A, Mulligan D, Rakela J, Douglas DD (2004). Hepatitis C virus recurrence in living donor liver transplant recipients. Dig Dis Sci.

[28] Thuluvath PJ, Yoo HY (2004). Graft and patient survival after adult live donor liver transplantation compared to a matched cohort who received a deceased donor transplantation. Liver Transpl.

[29] Van Vlierberghe H, Troisi R, Colle I, Ricciardi S, Praet M, de Hemptinne B (2004). Hepatitis C infection-related liver disease: patterns of recurrence and outcome in cadaveric and living-donor liver transplantation in adults. Transplantation.

[30] Terrault NA, Shiffman ML, Lok AS, Saab S, Tong L, Brown RS,Jr, Everson GT, Reddy KR, Fair JH, Kulik LM, Pruett TL, Seeff LB, A2ALL Study Group (2007). Outcomes in hepatitis C virus-infected recipients of living donor vs. deceased donor liver transplantation. Liver Transpl.

[31] Olthoff KM, Merion RM, Ghobrial RM, Abecassis MM, Fair JH, Fisher RA, Freise CE, Kam I, Pruett TL, Everhart JE, Hulbert-Shearon TE, Gillespie BW, Emond JC, A2ALL Study Group. (2005). Outcomes of 385 adult-to-adult living donor liver transplant recipients: a report from the A2ALL Consortium. Ann Surg.

[32] Broelsch CE, Frilling A, Testa G, Cicinnati V, Nadalin S, Paul A, Malago M (2003). Early and late complications in the recipient of an adult living donor liver. Liver Transpl.

[33] Schemmer P, Mehrabi A, Friess H, Sauer P, Schmidt J, Buchler MW, Kraus TW (2005). Living related liver transplantation: the ultimate technique to expand the donor pool?. Transplantation.

[34] Baltz AC, Trotter JF (2003). Living donor liver transplantation and hepatitis C. Clin Liver Dis.

[35] Marcos A, Fisher RA, Ham JM, Shiffman ML, Sanyal AJ, Luketic VA, Sterling RK, Fulcher AS, Posner MP (2000). Liver regeneration and function in donor and recipient after right lobe adult to adult living donor liver transplantation. Transplantation.

[36] Kam I (2002). Adult-adult right hepatic lobe living donor liver transplantation for status 2a patients: too little, too late. Liver Transpl.

[37] Liu LU, Bodian CA, Gondolesi GE, Schwartz ME, Emre S, Roayaie S, Schiano TD (2005). Marked Differences in acute cellular rejection rates between living-donor and deceased-donor liver transplant recipients. Transplantation.

[38] Takatsuki M, Uemoto S, Inomata Y, Egawa H, Kiuchi T, Fujita S, Hayashi M, Kanematsu T, Tanaka K (2001). Weaning of immunosuppression in living donor liver transplant recipients. Transplantation.

[39] Cotler SJ, Gaur LK, Gretch DR, Wile M, Strong DM, Bronner MP, Carithers RLJr, Emond MJ, Perkins JD, Nelson KA (1998). Donor-recipient sharing of HLA class II alleles predicts earlier recurrence and accelerated progression of hepatitis C following liver transplantation. Tissue Antigens.

[40] Manez R, Mateo R, Tabasco J, Kusne S, Starzl TE, Duquesnoy RJ (1995). The influence of HLA donor-recipient compatibility on the recurrence of HBV and HCV hepatitis after liver transplantation. Transplantation.

[41] Agnello V, Abel G, Elfahal M, Knight GB, Zhang QX (1999). Hepatitis C virus and other flaviviridae viruses enter cells via low density lipoprotein receptor. Proc Natl Acad Sci U S A.

[42] Monazahian M, Böhme I, Bonk S, Koch A, Scholz C, Grethe S, Thomssen R (1999). Low density lipoprotein receptor as a candidate receptor for hepatitis C virus. Med Virol.

[43] Gaglio PJ, Malireddy S, Levitt BS, Lapointe-Rudow D, Lefkowitch J, Kinkhabwala M, Russo MW, Emond JC, Brown RSJr (2003). Increased risk of cholestatic hepatitis C in recipients of grafts from living versus cadaveric liver donors. Liver Transpl.

[44] Garcia-Retortillo M, Forns X, Llovet JM, Navasa M, Feliu A, Massaguer A, Bruguera M, Fuster J, Garcia-Valdecasas JC, Rimola A (2004). Hepatitis C recurrence is more severe after living donor compared to cadaveric liver transplantation. Hepatology.

[45] Shiffman ML, Stravitz RT, Contos MJ, Mills AS, Sterling RK, Luketic VA, Sanyal AJ, Cotterell A, Maluf D, Posner MP, Fisher RA (2004). Histologic recurrence of chronic hepatitis C virus in patients after living donor and deceased donor liver transplantation. Liver Transpl.

[46] Sheiner PA, Boros P, Klion FM, Thung SN, Schluger LK, Lau JY, Mor E, Bodian C, Guy SR, Schwartz ME, Emre S, Bodenheimer HCJr, Miller CM (1998). The efficacy of prophylactic interferon alfa-2b in preventing recurrent hepatitis C after liver transplantation. Hepatology.

[47] Yedibela S, Schuppan D, Muller V, Schellerer V, Tannapfel A, Hohenberger W, Meyer T (2005). Successful treatment of hepatitis C reinfection with interferon-alpha2b and ribavirin after liver transplantation. Liver Int.

[48] Abdelmalek MF, Firpi RJ, Soldevila-Pico C, Reed AI, Hemming AW, Liu C, Crawford JM, Davis GL, Nelson DR (2004). Sustained viral response to interferon and ribavirin in liver transplant recipients with recurrent hepatitis C. Liver Transpl.

[49] Charlton M, Seaberg E, Wiesner R, Everhart J, Zetterman R, Lake J, Detre K, Hoofnagle J (1998). Predictors of patient and graft survival following liver transplantation for hepatitis C. Hepatology.

[50] Nelson DR, Soldevila-Pico C, Reed A, Abdelmalek MF, Hemming AW, Van der Werf WJ, Howard R, Davis GL (2001). Anti-interleukin-2 receptor therapy in combination with mycophenolate mofetil is associated with more severe hepatitis C recurrence after liver transplantation. Liver Transpl.

[51] Charlton M, Seaberg E (1999). Impact of immunosuppression and acute rejection on recurrence of hepatitis C: results of the National Institute of Diabetes and Digestive and Kidney Diseases Liver Transplantation Database. Liver Transpl Surg.

[52] Maluf DG, Stravitz RT, Cotterell AH, Posner MP, Nakatsuka M, Sterling RK, Luketic VA, Shiffman ML, Ham JM, Marcos A, Behnke MK, Fisher RA (2005). Adult living donor versus deceased donor liver transplantation: a 6-year single center experience. Am J Transplant.

[53] Eason JD, Nair S, Cohen AJ, Blazek JL, Loss GE,Jr (2003). Steroid-free liver transplantation using rabbit antithymocyte globulin and early tacrolimus monotherapy. Transplantation.

[54] Berenguer M, Prieto M, Palau A, Rayon JM, Carrasco D, Juan FS, López-Labrador FX, Moreno R, Mir J, Berenguer J (2003). Severe recurrent hepatitis C after liver retransplantation for hepatitis C virus-related graft cirrhosis. Liver Transpl.

[55] Ghobrial RM, Farmer DG, Baquerizo A, Colquhoun S, Rosen HR, Yersiz H, Markmann JF, Drazan KE, Holt C, Imagawa D, Goldstein LI, Martin P, Busuttil RW (1999). Orthotopic liver transplantation for hepatitis C: outcome, effect of immunosuppression, and causes of retransplantation during an 8-year single-center experience. Ann Surg.

[56] Rosen HR, Martin P (1998). Hepatitis C infection in patients undergoing liver retransplantation. Transplantation.

[57] Facciuto M, Heidt D, Guarrera J, Bodian CA, Miller CM, Emre S, Guy SR, Fishbein TM, Schwartz ME, Sheiner PA (2000). Retransplantation for late liver graft failure: predictors of mortality. Liver Transpl.

[58] Sheiner PA, Schluger LK, Emre S, Thung SN, Lau JY, Guy SR, Schwartz ME, Miller CM (1997). Retransplantation for recurrent hepatitis C. Liver Transpl Surg.

[59] Testa G, Crippin JS, Netto GJ, Goldstein RM, Jennings LW, Brkic BS, Brooks BK, Levy MF, Gonwa TA, Klintmalm GB (2000). Liver transplantation for hepatitis C: recurrence and disease progression in 300 patients. Liver Transpl.

[60] Carithers RL,Jr (1997). Recurrent hepatitis C after liver transplantation. Liver Transpl Surg.

[61] Dickson RC, Caldwell SH, Ishitani MB, Lau JY, Driscoll CJ, Stevenson WC, McCullough CS, Pruett TL (1996). Clinical and histologic patterns of early graft failure due to recurrnet hepatitis C in four patients after liver transplantation. Transplantation.

[62] Ghobrial RM, Colquhoun S, Rosen H, Hollis P, Ponthieux S, Pakrasi A, Farmer DG, Markman JF, Markowitz J, Drazan K, Yersiz H, Singer J, Stribling R, Arnout W, Holt CD, Goss J, Imagawa D, Seu P, Goldstein LI, Shackleton CR, Martin P, Busuttil RW (1998). Retransplantation for recurrent hepatitis C following tacrolimus or cyclosporine immunosuppression. Transplant Proc.

[63] Sarasin FP, Majno PE, Llovet JM, Bruix J, Mentha G, Hadengue A (2001). Living donor liver transplantation for early hepatocellular carcinoma: A life-expectancy and cost-effectiveness perspective. Hepatology.

[64] Pomfret EA, Washburn K, Wald C, Nalesnik MA, Douglas D, Russo M, Roberts J, Reich DJ, Schwartz ME, Mieles L, Lee FT, Florman S, Yao F, Harper A, Edwards E, Freeman R, Lake J (2010). Report of a national conference on liver allocation in patients with hepatocellular carcinoma in the United States. Liver Transpl.

[65] Lai JC, Pichardo EM, Emond JC, Brown RS,Jr (2009). Resource utilization of living donor versus deceased donor liver transplantation is similar at an experienced transplant center. Am J Transplant.

[66] Northup PG, Abecassis MM, Englesbe MJ, Emond JC, Lee VD, Stukenborg GJ, Tong L, Berg CL, Adult-to-Adult Living Donor Liver. Transplantation Cohort Study Group. (2009). Addition of adult-to-adult living donation to liver transplant programs improves survival but at an increased cost. Liver Transpl.

